# A Co-delivery System Based on a Dimeric Prodrug and Star-Shaped Polymeric Prodrug Micelles for Drug Delivery

**DOI:** 10.3389/fchem.2021.765021

**Published:** 2021-10-22

**Authors:** Man Zhou, Yan Luo, Weijia Zeng, Xiaoqing Yang, Tingting Chen, Lulu Zhang, Xiaoyan He, Xiuguang Yi, Yongxiu Li, Xiaoqing Yi

**Affiliations:** ^1^ College of Chemistry, School of Pharmacy, Nanchang University, Nanchang, China; ^2^ Key Laboratory of Prevention and Treatment of Cardiovascular and Cerebrovascular Diseases, Ministry of Education, Key Laboratory of Biomaterials and Biofabrication in Tissue Engineering of Jiangxi Province, College of Pharmacy, Gannan Medical University, Ganzhou, China; ^3^ School of Life Sciences, Anhui Medical University, Hefei, China; ^4^ School of Chemistry and Chemical Engineering, Jinggangshan University, Ji’an, China

**Keywords:** drug delivery, dimeric prodrug, polymeric prodrug micelles, high drug loading, reduction-sensitive

## Abstract

Chemotherapy is one of the commonly used therapies for the treatment of malignant tumors. Insufficient drug-loading capacity is the major challenge for polymeric micelle–based drug delivery systems of chemotherapy. Here, the redox-responsive star-shaped polymeric prodrug (PSSP) and the dimeric prodrug of paclitaxel (PTX) were prepared. Then the dimeric prodrug of PTX (diPTX, diP) was loaded into the core of the star-shaped polymeric prodrug micelles of PSSP by hydrophobic interaction forming the redox-responsive prodrug micelles of diPTX@PSSP for intracellular drug release in tumor cells. The hydrodynamic diameter of diPTX@PSSP nanoparticles was 114.3 nm ± 2.1 (PDI = 0.219 ± 0.016), and the micelles had long-term colloidal stability and the drug-loading content (DLC) of diPTX and PTX is 16.7 and 46.9%, respectively. The prepared micelles could broke under the reductive microenvironment within tumor cells, as a result, the dimeric prodrug of diP and polymeric prodrug micelles of PSSP were rapidly disassembled, leading to the rapid release of intracellular drugs. *In vitro* release studies showed that under the condition of reduced glutathione (GSH) (10 mM), the release of PTX was significantly accelerated with approximately 86.6% released within 21 h, and the released PTX in cytoplasm could promote the disintegration of microtubules and induce cell apoptosis. These results indicated that the new type of this reduction-sensitive nanodrug delivery system based on dimeric prodrug@polymeric prodrug micelles would be a promising technology in chemotherapy.

## Introduction

Polymeric micelles with a well-defined core-shell structure, which can improve the solubility, bioavailability, and circulation half-life of hydrophobic drugs thus became a promising nano-carrier for cancer treatment (Feng et al., 2020; Gauger et al., 2020; Yi et al., 2021a). Due to the unique physiological property of tumor blood vessels, polymer micelles usually from 50 to 150 nm can be passively enriched in tumor tissues through the enhanced permeability and retention (EPR) effect (Yi et al., 2021b; Ghosh and Biswas, 2021; Kaur et al., 2021). Although nano-sized polymeric micelles can efficiently accumulate in tumor tissues, insufficient drug loading is still one of the main challenges for polymeric micelles in the drug delivery system.

The prodrug is obtained by directly coupling the drug and the carrier through chemical bonds, which can accurately control the drug loading and increase the solubility and stability of the drug (Deng et al., 2021; Du et al., 2021; Yang et al., 2021). It thus changes the biodistribution, improves pharmacokinetics and pharmacodynamics, increases therapeutic effect, and reduces side effect (Dhiman et al., 2021; Wang et al., 2021). FDA-approved polymers such as PLGA, PEG, and dextran have been widely used in the development of polymer-drug conjugates (prodrugs) (Li et al., 2017; Hong et al., 2020; Zeng et al., 2020; An et al., 2021). The therapeutic effect of nano-drug not only depends on the enrichment effect of nanoparticles in tumor tissues, but also on the exposure amount of free drugs. In order to achieve controlled drug release in tumor cells, many efforts have been devoted to introduction of chemical bonds between drugs and polymers, which are the intrinsic stimuli of the tumor microenvironment, such as acidity, redox potential, and specific enzymes (Chakroun et al., 2020; Hao et al., 2020; Sun and Zhong, 2020; Uthaman et al., 2020). For redox potential, the concentration of glutathione (GSH) in tumor cells is much higher than that in the extracellular. Among them, the concentration of tumor cells is much higher than that in the normal cells (∼2 μmol) (Tang et al., 2020). The disulfide bonds introduced in the polymeric prodrug micelles can be reduced at high levels of GSH in the cytoplasm, leading to the dissociation of the carriers and subsequent intracellular release of the drug (Chen et al., 2018; Wang et al., 2020). Therefore, designing reduction-sensitive disulfide bonds as bridge bonds to construct polymeric prodrug nanoparticles is a common strategy for drug delivery systems.

Moreover, polymer prodrugs can also be used as drug carriers to load other drugs for combination therapy (Yi et al., 2016; Lu et al., 2020). It is worth noting that the drug loading and encapsulation efficiency of small hydrophobic drugs loaded by amphiphilic polymers are usually low (less than 10%) (Shen et al., 2017; Wang et al., 2017). This result may be mainly attributed to the formation of large drug aggregates through the packing of drug molecules with a long-range order (Cai et al., 2015). The design of the dimeric prodrug has emerged as one of the new potential strategies for increasing drug loading by hydrophobic interactions, due to the fact that drug dimers can prevent large particle formation (He et al., 2018). Furthermore, the dimeric prodrug possess the stronger intermolecular hydrophobic interactions than the free drug because of the increased surface area and the enhanced tendency of the prodrug to aggregate (Pei et al., 2018; Li et al., 2020; Zuo et al., 2020). Therefore, the design of the drug delivery system based on dimeric prodrug can improve drug loading effectively.

**SCHEME 1:**
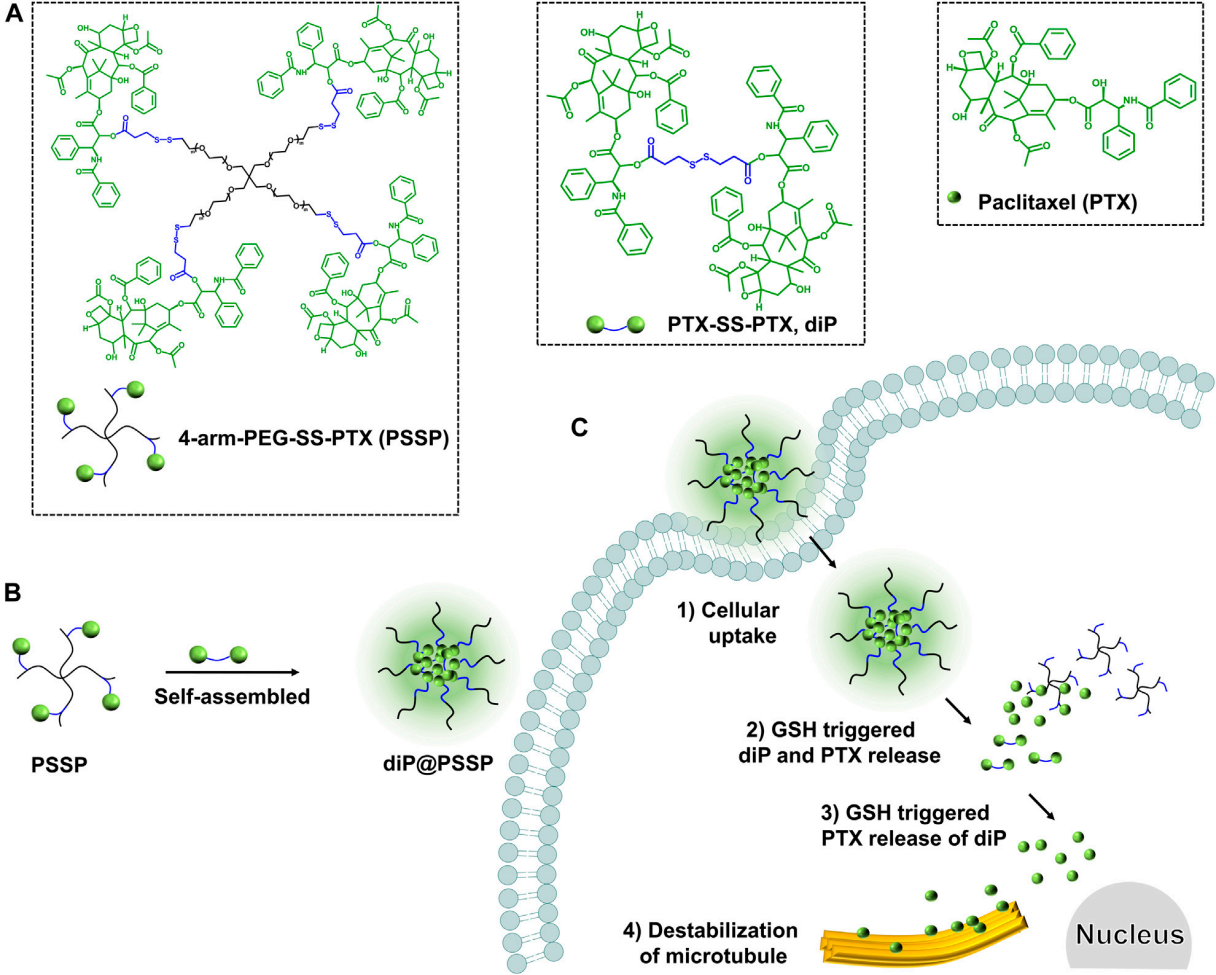
A reduction-sensitive nanodrug delivery system based on dimeric prodrug@polymeric prodrug micelles for drug delivery. **(A)** The structure of the star-shaped polymeric prodrug (4-arm PEG-SS-PTX, PSSP) and reduction-sensitive dimer-PTX (PTX-SS-PTX, diP) with disulfide linker. **(B)** diP was encapsulated into the core of PSSP micelles to obtain diP@PSSP micelles by hydrophobic effect. **(C)** PTX release mechanism of diP@PSSP micelles in tumor cells.

In order to obtain the nanodrug delivery system with a high drug-loading content (DLC), in this study, a prodrug micelle with DLC composed of a redox-responsive star-shaped polymeric prodrug of paclitaxel (PTX) and a redox-responsive dimeric prodrug of PTX was established to inhibit the growth of tumor cells. As shown in Scheme 1, the star-shaped polymeric prodrug (4-arm-PEG-SS-PTX, PSSP) and the reduction-sensitive dimer-PTX (PTX-SS-PTX, diP) with disulfide linker were synthesized by a thiol-disulfide exchange reaction and an esterification reaction, respectively. Then the dimeric prodrug of diP was loaded into the hydrophobic core of the polymeric prodrug of PSSP micelles by the hydrophobic interaction to obtain diP@PSSP micelles. The diP@PSSP micelle has good stability, the size of the diP@PSSP micelle is 114.3 nm ± 2.1 (PDI = 0.219 ± 0.016), and the DLC of diPTX and PTX is 16.7 and 46.9%, respectively. This diP@PSSP micelle is specifically internalized into tumor cells by EPR effect and the disulfide bond could be cleaved immediately under the condition of high concentration of GSH, thereby releasing PTX and diPTX. Subsequently, the released diPTX and PTX could also be released from the dimeric prodrug of diP. Finally, the released drug would disrupt the balance of microtubule polymerization and depolymerization to inhibit the growth of tumor cells.

## Results and Discussion

### Characterization of the Prepared Prodrug

**FIGURE 1:**
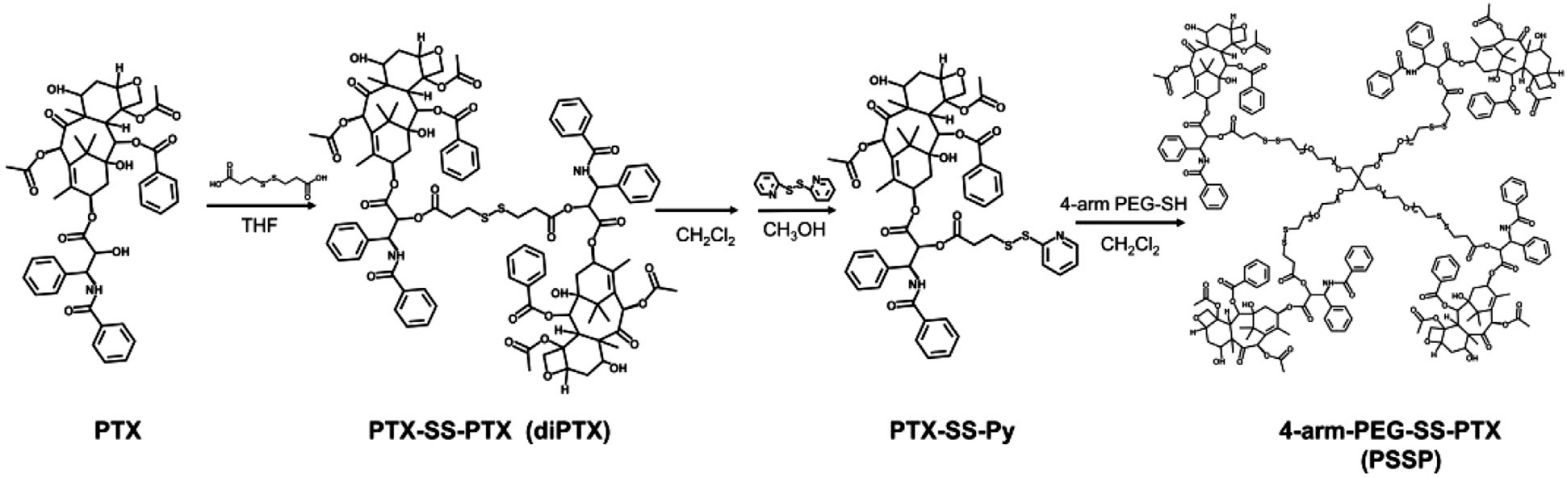
Facile synthesis of the redox-sensitive dimeric prodrug of dimer-PTX (PTX-SS-PTX, diP) and the star-shaped polymeric prodrug of 4-arm PEG-SS-PTX (PSSP).

**FIGURE 2:**
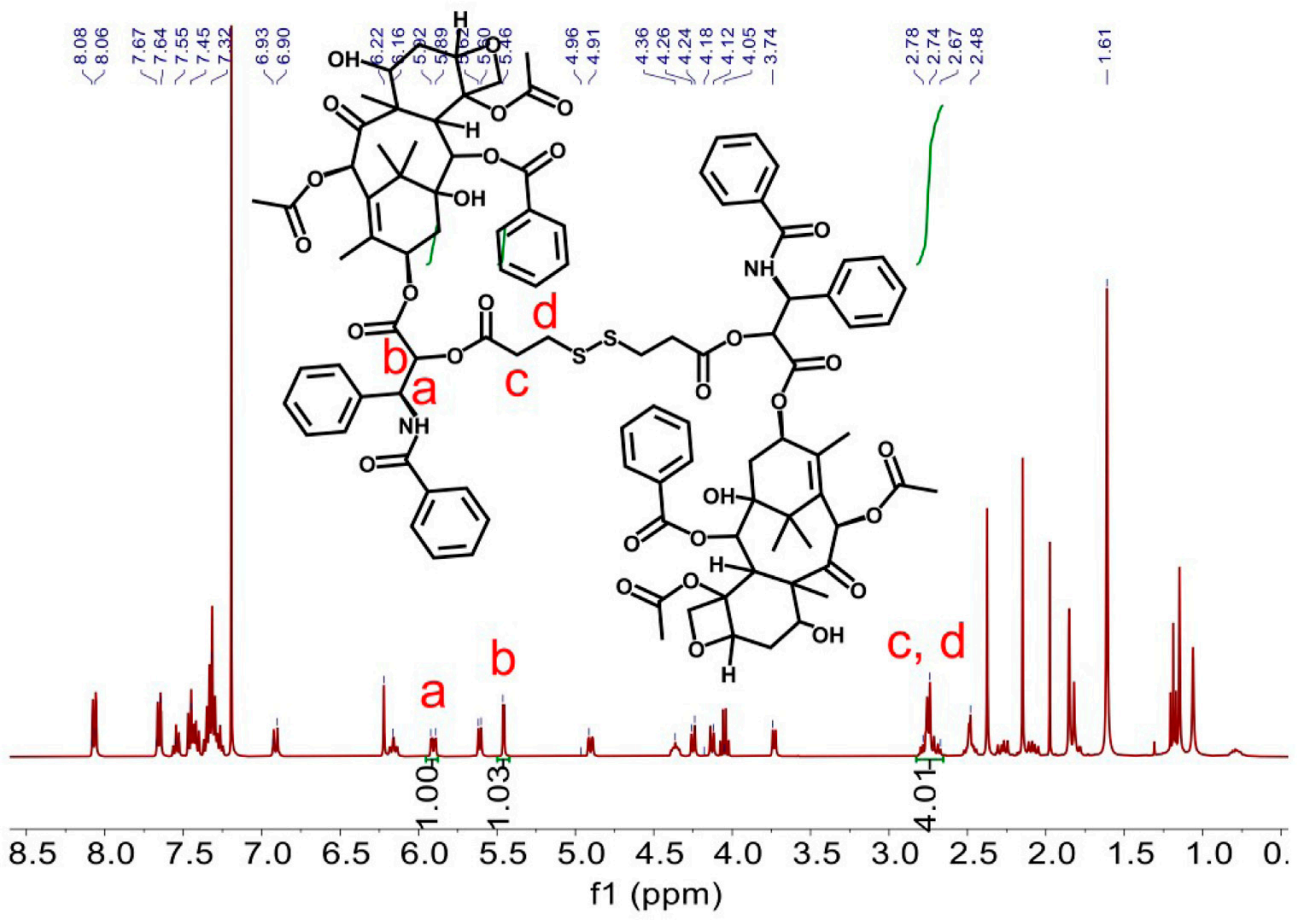
^1^H NMR spectrum (400 MHz, CDCl_3_) of diPTX.

**FIGURE 3:**
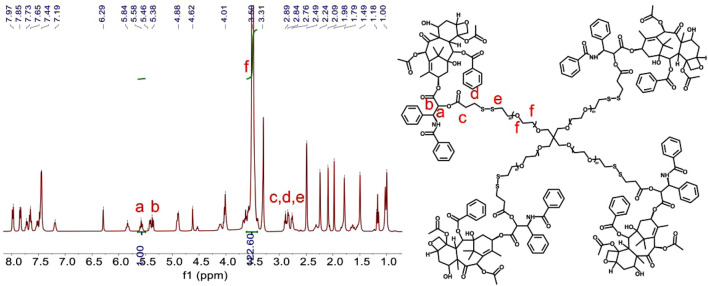
^1^H NMR spectrum (400 MHz, *d*-DMSO) of PSSP.

Redox-sensitive disulfides were introduced into the dimer prodrug and polymer prodrug to control drug release, the synthetic route of the redox-sensitive dimeric prodrug of dimer-PTX (PTX-SS-PTX, diP), and the star-shaped polymeric prodrug (4-arm-PEG-SS-PTX, PSSP) was shown in Figure 1A. The PTX reacted with 3,3′-dithiobispropionic acid to obtain the prodrug of diP by the esterification reaction (Yi et al., 2021a). Characterization was obtained by ^1^H NMR, The signal at δ 2.5–2.8 is assigned to the methylene proton beside the disulfide bond and the ester group in deuterated dichloromethane, and the integral ratio of δ 2.5–2.8 is about four times than that of δ 5.46, which shows the successful synthesis of the dimeric prodrug of diP (Figure 2). Next, diP was treated with DTT to afford PTX-SH by the redox reaction and then PTX-SH reacted with excess 2,2′- dipyridyl disulfide to obtain the product of pyridyl disulfide-PTX (PTX-SS-Py) by the thiol-exchange reaction. The ^1^H NMR spectrum of PTX-SS-Py was shown in Supplementary Figure S1; the shift at δ 8.71 was attributed to methyne proton of the pyridyl disulfide group and the integral of δ 8.71 and δ 5.85 is close to 1:1, and the mass data of PTX-SS-Py was 1,051.3357; the result was close to the theoretical value (calcd [M + H]^+^ = 1,051.3351) (Supplementary Figure S2), which shows the successful synthesis of the PTX-SS-Py. Finally, the star-shaped polymeric prodrug of PSSP was synthesized *via* the thiol-exchange reaction between 4-arm PEG-SH and PTX-SS-Py. The characteristic peaks of PTX-SS-Py units (δ 5.54, 5.46, 5.38, 2.89, 2.84, and 2.76) and PEG units (δ 3.50, 3.31) were observed for PSSP (Figure 3). Moreover, the intensity ratio of the two signals at δ 5.58 and δ 3.50 on PTX units and PEG units, respectively, is close to 1:124, indicating that the PSSP with a four-armed star-shaped amphiphilic polymer was prepared successfully. The above shows that the redox-sensitive dimeric prodrug of diP and the star-shaped polymeric prodrug of PSSP were synthesized successfully.

### Characterization of the Micelles

**FIGURE 4:**
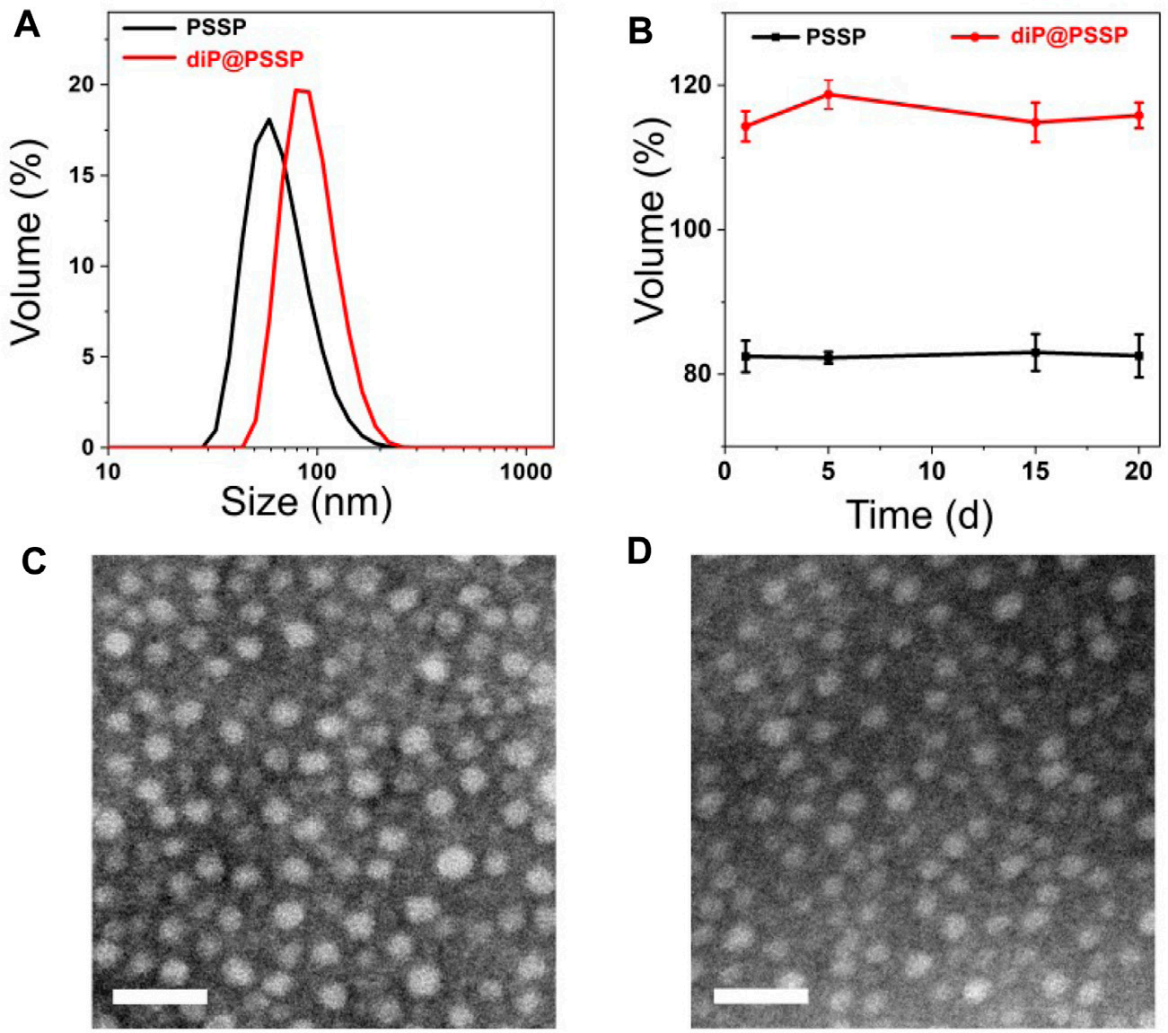
**(A)** Hydrodynamic size distribution of PSSP and diP@PSSP micelles. **(B)** Stability assay of PSSP and diP@PSSP micelles in PBS during 20-day storage at room temperature. The TEM images of **(C)** PSSP and **(D)** diP@PSSP micelles. The scale bar is 100 nm.

**FIGURE 5:**
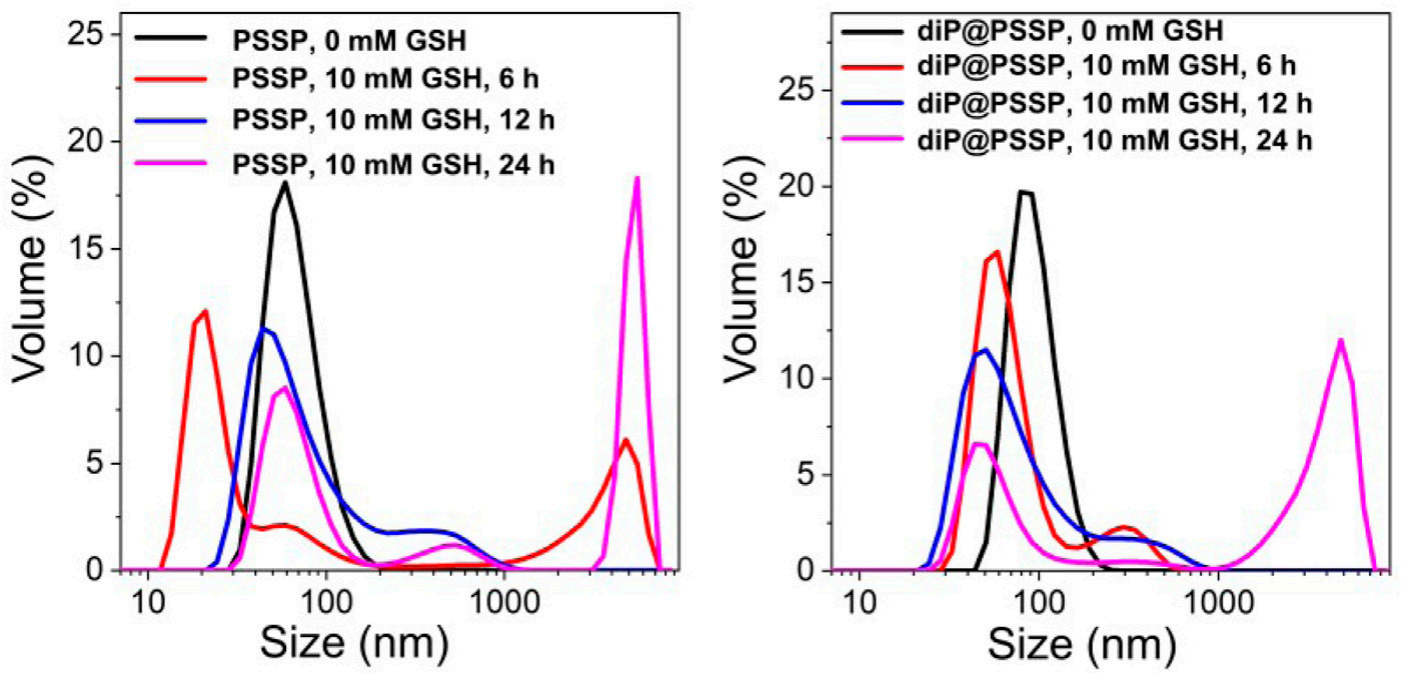
The size changes of **(A)** PSSP and **(B)** diP@PSSP micelles in response to 10 mM GSH at pH 7.4 determined by DLS, respectively.

The hydrophobic dimeric prodrug of diP was encapsulated in the polymeric prodrug micelles of PSSP to prepare diP@PSSP micelles. The DLC of diP and PTX were determined and calculated by high-performance liquid chromatography (HPLC) to be 16.7 and 46.9%, respectively. The size of micelles is one of the key parameters affecting their stability *in vivo*. The average diameters of the prepared PSSP and diP@PSSP micelles were 83.1 ± 0.4 nm (PDI = 0.124 ± 0.016) and 114.3 ± 2.1 nm (PDI = 0.219 ± 0.016), respectively, determined by the dynamic light scattering (DLS) (Figure 4A). The PSSP and diP@PSSP micelles have good stability, while their size remained essentially unchanged during 20-day storage at room temperature (Figure 4B). The morphology of PSSP and diP@PSSP micelles were investigated by a transmission electron microscope (TEM). The micelles showed spherical morphology and the sizes were smaller than the micelles obtained by DLS, which might be caused by the collapse of the hydrophilic PEG shell in the dry state (Wu et al., 2020) (Figures 4C,D). This size of micelles ranged from 50 to 150 nm could passively target the tumor with high efficiency through EPR effect (Kang et al., 2020; Kuang et al., 2020). Then the character of the reduction-sensitive was verified in this drug delivery system, DLS was used to monitor the size change of PSSP and diP@PSSP micelles in response to 10 mM GSH (Figure 5). The results showed that the PSSP and diP@PSSP micelles were quickly destabilized by GSH to form smaller and larger aggregates simultaneously within 6 h, the particle size distribution continued to change with the increase of time. This may be due to GSH triggering the cleavage of the disulfide bond of PSSP and diP to form water-soluble PEG and release water-insoluble PTX. The above results indicate that PSSP and diP@PSSP micelles were prepared successfully by self-assembly, and the PSSP and diP@PSSP micelles have good size distribution and excellent reduction responsiveness.

### Drug Release and Cytotoxicity Assay

**FIGURE 6:**
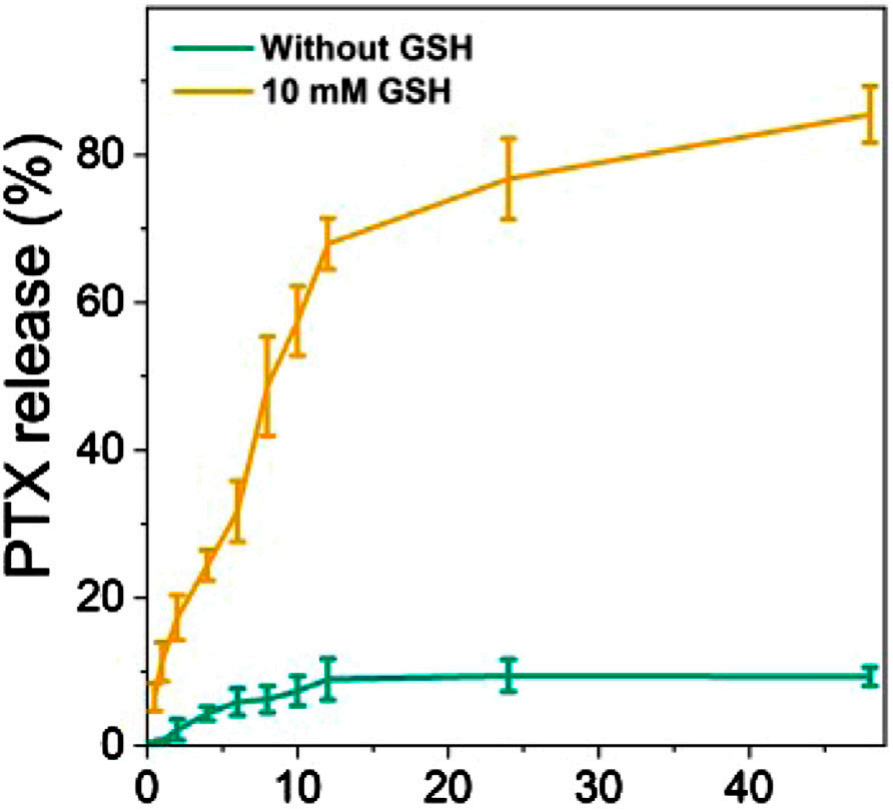
PTX was released from a PBS solution of diP@PSSP micelles containing 0.1% (w/v) Tween 80 (pH 7.4, 0.1 M) at 37°C, with or without 10 mM GSH.

**FIGURE 7:**
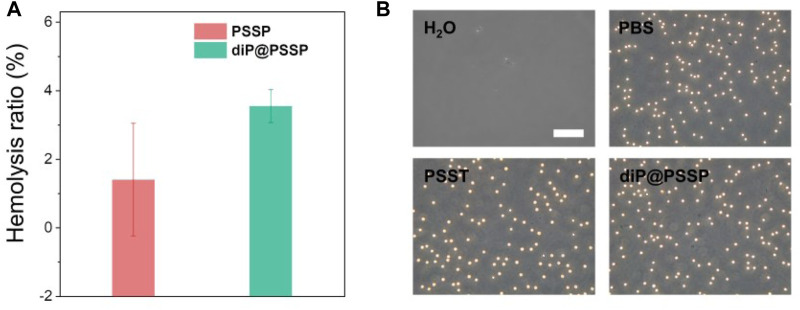
**(A)** The hemolysis ratio induced by PSSP and diP@PSSP micelles with the concentration of 200 μg ml^−1^ incubated at 37°C for 6 h in dark. **(B)** Optical microscopic observation of the dispersion states of the RBCs after incubated with H_2_O, PBS, PSSP, and diP@PSSP micelles for 6 h. Scale bar: 50 μm.

The PTX release behaviors of the diP@PSSP micelles in reduction environment of 10 mM GSH were evaluated *in vitro*, the results were shown in Figure 6. Only less than 10% of the PTX was cumulative released at pH 7.4 in absence of 10 mM GSH within 48 h, which simulates the physicochemical environment of blood circulation. This indicates that the micelle of diP@PSSP have good stability to inhibit premature drug leakage, which may be due to the hydrophobic interaction between PTX and diPTX and the stable disulfide bond of the conjugated PTX (Yang et al., 2019). However, a significant increase in drug release was observed at the condition of 10 mM GSH. The cumulative drug release of diP@PSSP micelles was about 85% within 48 h. These results show that the disulfide bonds collapse occurred under the activation of GSH, which can accelerate the release of PTX from diP@PSSP micelles (Su et al., 2018). In order to verify the prepared prodrug micelles in a short period of time is not seriously destructive to red blood cells (RBCs), the hemolysis of PSSP and diP@PSSP micelles was studied (Chibhabha et al., 2020). As shown in Figure 7A, the prepared micelles had no obvious corrosive effect on the behavior of RBCs. In addition, PSSP and diP@PSSP micelles exerted neglectable hemolysis (<5.0%) for RBCs at the concentration of 200 μg ml^−1^ (Figure 7B). The results show that the prepared PSSP and diP@PSSP micelles were safe drug delivery systems with good biocompatibility.

**FIGURE 8:**
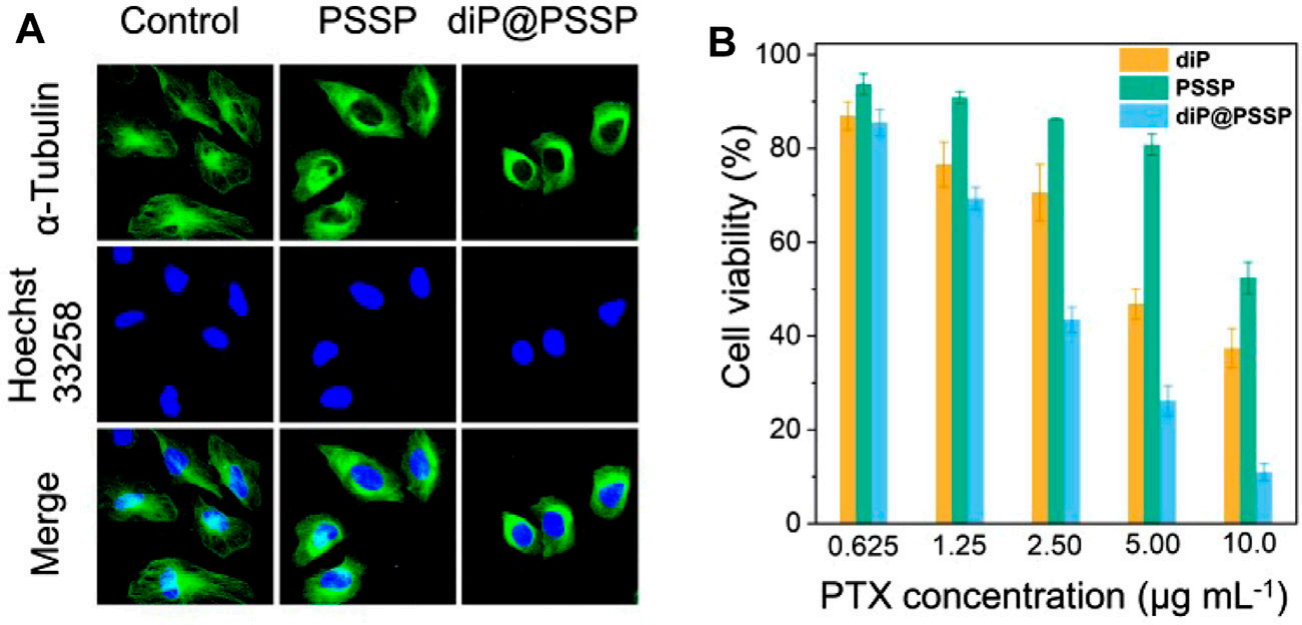
**(A)** Detection of microtubules in HeLa cells after incubation with PSSP and diP@PSSP micelles for 8 h, respectively. **(B)** Viability of HeLa cells treated with diP, PSSP, and diP@PSSP nanoparticles under different concentration for 48 h, respectively. Scale bar: 20 μm.

It is well known that PTX is a hydrophobic drug that induces apoptosis in tumor cells by promoting microtubule disintegration. Microtubule is a kind of natural biological macromolecule existing in cells, which can continuously change its assembly and disassembly state in a few seconds in life activities (Chan and Coen, 2020). In order to studied the effect of polymeric prodrug PSSP and diP@PSSP micelles on microtubules of HeLa cells, the morphology of microtubules was observed by CLSM after the HeLa cells were incubated with the PSSP and diP@PSSP micelles for 8 h. As shown in Figure 8A, compared with the control group, PSSP and diP@PSSP micelles showed significant contraction effect on the microtubules of HeLa cells. This is due to the presence of PTX in PSSP and diP@PSSP micelles, and the PTX released under reduction condition in tumor microenvironment can bind to specific sites of tubulin to prevent its depolymerization. To study the cytotoxicity of the diP, PSSP, and diP@PSSP micelles, the HeLa cells were incubated with these samples at the concentrations of 1.25, 2.50, and 5.00 μg ml^−1^ for 48 h. As shown in Figure 8B, the diP, PSSP, and diP@PSSP micelles exhibited toxicity in HeLa cells, indicating the tumor cells are very sensitive to these redox-responsive prodrugs. The inhibitory effect of PSSP micelles on HeLa cells growth was weaker than that of diP, which might be related to the protective shell of PEG. Moreover, the cell viability of diP@PSSP on HeLa cells is weaker than that of diP, which may be attributed to two reasons, one is that the solubility of diP is enhanced by PSSP carrier to facilitate its phagocytosis by cells and the other is that the released PTX in tumor environment exerts its drug effect. In general, diP@PSSP micelles with high drug loading not only have good biocompatibility but also can achieve controlled drug release in tumor cells and inhibit the growth of tumor cell.

## Conclusion

In summary, a new type of drug delivery system of diP@PSSP with high DLC and excellent stability was developed based on prodrug–drug complexes and dimeric prodrug. The polymeric prodrug diP@PSSP micelles possessed high DLC of PTX as high as 46.9% and excellent stability. The controlled release of PTX under the 10 mM GSH could be achieved for reductive-sensitive diP@PSSP micelles. In addition, the polymeric prodrug of diP@PSSP micelles showed good biocompatibility in RBCs and therapeutic effect in HeLa cells. We hope that this strategy will be used to facilitate the provision of multiple therapeutic drugs for a variety of purposes, in particular to overcome multidrug resistance.

## Data Availability

The original contributions presented in the study are included in the article/Supplementary Material; further inquiries can be directed to the corresponding authors.
