# Supplementary material for: A Co-delivery System Based on a Dimeric Prodrug and Star-Shaped Polymeric Prodrug Micelles for Drug Delivery
Source: Front Chem. 2021 Oct 22;9:765021. doi: 10.3389/fchem.2021.765021 (PMC8569866; doi:10.3389/fchem.2021.765021)
Supplement: Supplementary file 1 [file DataSheet2.PDF]

A co-delivery system based on a dimeric prodrug and star-shaped polymeric prodrug micelles for drug delivery

Man Zhou,[a,b] *Yan, Luo,*[a,b] *Weijia Zeng,*[b] *Xiaoqing Yang,*[b] *Tingting Chen,*[b] *Lulu Zhang,*[b] *Xiaoyan He,*[c,*] *Xiuguang Yi,*[d] *Yongxiu Li,*[*,a] *and Xiaoqing Yi,*[*,b]

[a] College of Chemistry, School of pharmacy, Nanchang University, Nanchang 341000, China.

[b] Key Laboratory of Prevention and Treatment of Cardiovascular and Cerebrovascular Diseases, Ministry of Education, Key Laboratory of Biomaterials and Biofabrication in Tissue Engineering of Jiangxi Province, College of Pharmacy, Gannan Medical University, Ganzhou 341000, China.

[c] School of Life Sciences, Anhui Medical University, Hefei 230032, China.

[d] School of Chemistry and Chemical Engineering, Jinggangshan University, Ji'an 343100, China.

* Corresponding author. E-mail: hexiaoyan@ahmu.edu.cn (X. He)
yxli@ncu.edu.cn (Y. Li)
keyi0115@126.com (X. Yi).

Experimental Details

Materials. 3,3′-dithiodipropionic acid, paclitaxel (PTX), DL-dithiothreitol (DTT), 2,2'-dithiodipyridine, dicyclohexylcarbodiimide (DCC), 4-dimethylaminopyridine (DMAP), DMSO and glutathione (GSH) were purchased from Macklin Co. Ltd. and used as received. 4 armed PEG-SH (M_W = 5000) were synthesized by Xi'an Ruixi Biological Technology Co. Ltd. and used as received. Fetal bovine serum (FBS), phosphate-buffered saline (PBS), Dulbecco's Modified Eagle's Medium (DMEM), trypsin and penicillin-streptomycin were purchased from Gibco and used as received.

Cell culture. HeLa cells were cultured in DMEM supplemented with 1% streptomycin, 1% penicillin and 10% fetal bovine serum (FBS) in 100% humidity and 5% CO_2 atmosphere at 37°C.

Characterizations. Bruker AM 400 apparatus was used to measure the ^{1}H nuclear magnetic resonance (^{1}H NMR) spectrum. The dynamic light scattering (DLS, Malvern Zetasizer Nano ZS90) was used to measure the average size of nanoparticles. Transmission electron microscopy (TEM, FEI Tecnai G20) was used to determine the morphology of nanoparticles. The drug release of diP@PSSP was determine by high-performance liquid chromatography (HPLC, Waters 2695e).

Synthesis of reduction-sensitive dimer prodrug (diPTX). PTX (2.00 g, 2.34 mmol), 3,3′-dithiodipropionic acid (246 mg, 1.17 mmol) and DMAP (28.6 mg, 0.234 mmol) were added in the CH_2Cl_2 and stirring for 2 h. Then, DCC (525 mg, 2.55 mmol) was added and reacted at 40°C for 3 day. Finally, the product of diPTX was purified by silica gel chromatography. The white solid product was obtained finally. The yield is 73.2%.

Synthesis of PTX-SS-Py. The mixture of diPTX (1.2 g, 0.638 mmol) and DTT (386 mg, 2.5 mmol) was stirred in dichloromethane under argon atmosphere at room temperature for 1 d. Then the reaction solution was concentrated and precipitated in ether twice to give the crude product of PTX-SH. To prepare PTX-SS-Py, the above crude product was stirred with 2,2'-dithiodipyridine (282 mg, 1.28 mmol) in methanol solution at room temperature for 1 d. Finally, the product of diPTX was purified by silica gel chromatography. The white solid product was obtained finally. The yield is 57.2%.

Synthesis of reduction-sensitive star-shaped polymeric prodrug (4-arm-PEG-SS-PTX, PSSP). Under an argon atmosphere, the synthesized PTX-SS-Py (336 mg, 0.32 mmol) and 4-arm-PEG-SH (0.40 g, 0.08 mmol) were stirred in dichloromethane solution for 3 day. Finally, the product was further purified by dialysis against DMSO and deionized water for 48 h, respectively. Finally, the product of PSS was obtained by freeze-dry to give a white solid in 92.4% yield.

Preparation of the micelle of PSSP and diP@PSSP. The PSSP and diP@PSSP micelles were prepared by the dialysis method. In brief, PSSP and diP was dissolved in DMF and then dialyzed against deionized water for 24 h. PSSP micelles are prepared in a similar manner.

pH-triggered change of micelle sizes. The size change of PSSP and diP@PSSP micelles in response to 10 mM GSH and acetate buffer (100 mM, pH 5.0) was monitored by dynamic light scattering (DLS) measurement. Briefly, the micellar solutions were shaken at 37 °C under a nitrogen atmosphere. At the desired time intervals, the change of micellar size was measured by DLS.

The PTX release profiles of diP@PSSP micelles. The release profiles of PTX from diP@PSSP micelles were studied at 37°C in PBS (10 mM, pH 7.4) containing 0.1% (w/v) Tween 80 with or without 10 mM GSH by dialysis method. 2 mL of release medium was replaced with an equal volume of fresh media at predetermined time intervals, and the release medium was freeze-dried to obtain the released PTX. The concentration of paclitaxel was determined by HPLC.

Detection of microtubules in cells. After HeLa cells were incubated with PSSP and diP@PSSP micelles for 8 h, respectively. Then, the cells were fixed with 4% paraformaldehyde, permeabilized with 0.1% Triton X-100 in PBS and stained with anti-α-tubulin-FITC at 37°C for 1 h. Confocal lasing scanning microscopic (CLSM) was used to

image microtubules (Ex: 488 nm, Em: 505-540 nm).

Cell viability assay. The cytotoxicity assessment was carried out in HeLa cells by using the MTT assay. 100 μL of cell suspension were seeded into each well of a 96-well plate and incubated at 37 °C with 5% CO_2 for 24 h. Then the cells were treated with the samples at various concentrations and a further incubation for 48 h was carried out. After that, 10 μL of MTT solution in PBS (5 mg mL^{-1}) was added into each well and the cells were incubated for another 4 h. The medium in each well was carefully removed and replaced by 100 μL DMSO. When the purple solution was homogeneous, the absorbance at 570 nm was recorded by using a microplate reader (Multiskan GO, Thermo Fisher). Cell viability was calculated by: Cell viability (%) = $(A_{\text{treated}} - A_0)/(A_{\text{control}} - A_0) \times 100\%$.

The assays were conducted in triplicate. The results are shown as the average value ± SD.

Hemolysis test. The hemolytic activity of PSSP and diP@PSSP micelles on mouse blood cells was tested by microplate reader. Briefly, a mouse blood sample was first taken, and 2% of red blood cells were prepared. Then, red blood cell suspension (0.2 mL) was incubated with physiological saline solution (0.2 mL), ultrapure water (0.2 mL), PSSP micelles (0.2 mL), and diP@PSSP micelles (0.2 mL) at 37°C for 6 h, respectively. Finally, microplate reader was used to measure these samples. Physiological saline solution and ultrapure water were used as negative control (causing no hemolysis) and positive control (causing 100% hemolysis), respectively. The hemolysis ratio of red blood cells was calculated using the following formula: hemolysis (%) = $(A_{\text{sample}} - A_{\text{negative}})/(A_{\text{positive}} - A_{\text{negative}}) \times 100\%$, where A_{sample}, A_{negative}, and A_{positive} refer to the absorption of material sample solution, negative control and positive control.

Data availability. Data supporting the findings of this study are available within this article and its Supplementary Information file, and from the corresponding author on reasonable request.

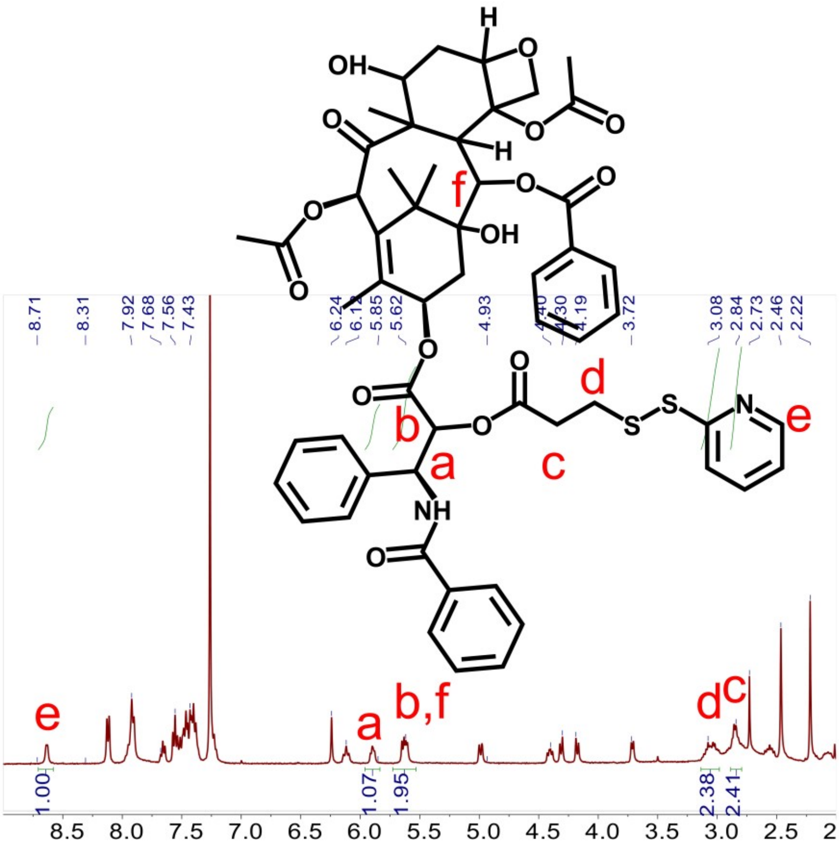

Figure S1. ^{1}H NMR spectrum (400 MHz, $CDCl_3$) of PTX-SS-Py.

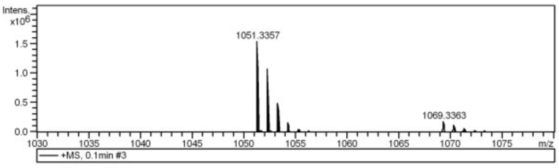

#	m/z	Res.	S/N	I	FWHM
1	1051.3357	22288	3249.0	1542937	0.0472
2	1052.3376	18975	2241.5	1066932	0.0555
3	1053.3330	14920	1038.8	495675	0.0706
4	1054.3284	8239	322.4	154204	0.1280
5	1055.3209	6176	105.2	50459	0.1709
6	1056.3141	7617	33.3	16026	0.1387
7	1057.3147	7423	8.8	4230	0.1424
8	1069.3363	9113	372.8	184943	0.1173
9	1070.3345	7896	245.9	122282	0.1356
10	1071.3368	6515	124.5	62059	0.1644
11	1072.3329	6095	48.9	24417	0.1759
12	1073.3119	5800	41.9	20969	0.1850
13	1074.3082	5778	22.6	11336	0.1859
14	1075.3029	7372	11.1	5581	0.1459

Figure S2. Mass spectrum of PTX-SS-Py, calcd $[M+H]^+$=1051.3351.
